# Salivary Gland Dysfunction Secondary to Cancer Treatment

**DOI:** 10.3389/froh.2022.907778

**Published:** 2022-06-09

**Authors:** Anette Vistoso Monreal, Gregory Polonsky, Caroline Shiboski, Vidya Sankar, Alessandro Villa

**Affiliations:** ^1^Department of Orofacial Sciences, University of California, San Francisco, San Francisco, CA, United States; ^2^General Practice Residency, Department of Oral and Maxillofacial Surgery, University of California, San Francisco, San Francisco, CA, United States; ^3^Department of Diagnostic Sciences, Tufts University, Boston, MA, United States

**Keywords:** cancer, dry mouth, xerostomia, hyposalivation, radiotherapy, chemotherapy, management

## Abstract

The number of cancer survivors are increasing and so are the oral toxicities from cancer therapy. Most patients receiving treatment for cancer develop some form of oral adverse events including, but not limited to, mucositis, opportunistic infections, dry mouth, and/or osteonecrosis of the jaw. One of the most common complications from head and neck cancer radiation therapy is salivary gland dysfunction (SGD). SGD is an umbrella term that includes the subjective sensation of dry mouth (xerostomia) and hyposalivation (objective reduction of the salivary flow rate). Dry mouth in cancer patients may lead to functional defects (e.g., eating, speaking, and swallowing), increase the risk of dental caries and oral candidiasis, and can have a negative effect on the nutritional and psychological status of the patients. The aim of this mini review was to summarize the current criteria for diagnosis and management of SGD associated with cancer treatment.

## Introduction

Cancer survival rates continues to increase and by 2040, it is estimated that there will be 26 million cancer survivors in the United States [1–3]. Cancer patients may develop acute and chronic oral toxicities from cancer therapy including mucositis, xerostomia, salivary gland dysfunction (SGD), neurosensory disorders, trismus, jaw necrosis, and infections to name a few. Cancer regimen-related toxicities often lead to devastating consequences, reduced function, poor clinical outcomes, and higher health care cost [4].

SGD is one of the most frequent side effects from cancer therapy [5]. Dry mouth in cancer patients may be secondary to chemotherapy, head and neck radiotherapy, dehydration, and chronic graft-vs. host disease (cGVHD). Saliva has important functions including antimicrobial activity, gustatory function, protection and lubrication of the oral mucosa and esophagus, and remineralization and maintenance of hard and soft tissues in the oral cavity; all these functions have the potential to be compromised by cancer therapy [6]. The aim of this mini review was to describe the established causes and guidelines for diagnosis and management options of SGD in cancer patients, as well as the potential new therapeutic approaches that are currently in study and development.

## Definitions and Diagnostic Tests For Salivary Gland Dysfunction

SGD has been defined as “any alteration in the qualitative or quantitative output of saliva caused by an increase (hyperfunction) or decrease (hypofunction) in salivary output” [7].

Hyposalivation is assessed by measuring stimulated and unstimulated salivary flow and at times, by individual major gland secretion. Hyposalivation is defined as a resting (unstimulated) whole saliva flow rate of ≤ 0.1 mL/min and/or a stimulated whole saliva flow rate of ≤ 0.5 mL/min [8, 9]. Commonly used stimulants to assess stimulated salivary flow rates include sugar free gums, paraffin wax, rubber bands, or citric acid. Hyposalivation may or may not result in xerostomia (the subjective feeling of dry mouth), negative impact on function, including eating, speaking, and swallowing, dysgeusia, and/or a burning sensation of the oral mucosa [10, 11].

Xerostomia in cancer patients is assessed by patient reported outcomes (PROs) [e.g., Xerostomia Inventory (XI)] or by practitioner reported outcomes [e.g., using the National Cancer Institute (NCI) Common Terminology Criteria for Adverse Events (PRO-CTCAE)], where in addition to the patient symptoms, the unstimulated saliva is measured (Table 1) [12, 13]. The XI is an 11-item instrument that evaluates and measures the different aspects of xerostomia that are experienced by the patient. A shortened version of XI named Summated Xerostomia Inventory (SXI) comprises five of the original 11 items and was more recently developed to focus on the “experiential aspects of dry mouth” rather than measuring general exocrine gland functions [14].

**Table 1:** Common Terminology Criteria for Adverse Events (CTCAE) for dry mouth.

**CTCAE term**	**Grade 1**	**Grade 2**	**Grade 3**	**Grade 4**	**Grade 5**
Dry mouth	Symptomatic (e.g., dry, or thick saliva) without significant dietary alteration; unstimulated saliva flow >0.2 ml/min	Moderate symptoms: oral intake alterations (e.g., copious water, other lubricants, diet limited to purees and/or soft, moist foods); unstimulated saliva 0.1 to 0.2 ml/min	Inability to adequately aliment orally; tube feeding or TPN indicated; unstimulated saliva <0.1 ml/min	*	*

*TNP, Total parental nutrition*.

*No applicable. Grade 4 refers to life-threatening consequences; urgent intervention indicated. Grade 5 refers death related to AE.

*The CTCAE displays Grades 1 through 5 with unique clinical descriptions of severity for each AE. Dry mouth is categorized under gastrointestinal disorders with Grade 1–3 severity of symptoms [12]*.

Excessive salivation is rare, and may occur in cancer patients due to dysphagia, odynophagia, malignancy, or local oral irritation. Non-pharmacologic treatment varies from functional dysphagia therapy to neurosensory approaches. Pharmacologic treatment aims to reduce salivary flow and includes several agents such as glycopyrrolate, scopolamine, atropine, benztropine, and botulinum toxin injection into the salivary glands [15]. Mucolytic agents such as guaifenesin and n-acetylcysteine may decrease the viscosity and volume of mucous secretions and improve comfort [16].

## SGD Secondary to Cancer Treatment

### Radiotherapy- Induced SGD

Dry mouth is one of the most common and dismal effects of radiation therapy (RT) to the head and neck cancer patients. Salivary gland tissue, in particular acinar cells of the serous glands (parotid), is sensitive to RT with permanent salivary gland damage in patients receiving cumulative doses > 30 Gy [17]. RT can also cause dry mouth due to indirect damage to epithelial and connective tissues of the gland including the blood vessels and nerves [4]. When salivary glands are within the field of radiation, irreversible salivary glands damage occurs in 63–93% of the patients [18]. In head and neck cancer patients undergoing RT, the dysfunction is dose dependent; when 40–50 Gy are administered up to 75% of the parotid gland function may be impaired [19]. The effects of RT on SGD are long term and usually irreversible [18].

Preventive strategies of salivary gland hypofunction and dry mouth secondary to RT have focused on the preservation of salivary gland function, primarily the parotid glands, by new advances in radiation techniques, including the appearance and optimizing of 3D treatment planning, conformal radiation techniques, and intensity-modified radiotherapy (IMRT) [20, 21]. Recent studies also showed that the prevention of hyposalivation secondary to RT may be addressed via use of cytoprotective agents (eg. amifostine), the application of lubricating or stimulatory agents, surgical transfer of submandibular glands, and acupuncture during and following cancer treatment [11, 22–24].

Treatment of salivary gland hypofunction secondary to RT includes systemic parasympathomimetic agents with muscarinic action (pilocarpine HCl and cevimeline) [25]. Pilocarpine is recommended to be administered at a dose of 5 mg 3 times a day for at least 3 months [26]. Cevimeline is also recommended for a minimum of 3 months to achieve clinical results, 30 mg 3 times daily [27]. Common side effects for both medications include increased excess of sweating, dyspepsia, nausea, and diarrhea [28]. Topical agents include over-the-counter saliva substitutes and mucosal lubricants, as well as non-pharmacological approaches to mechanically stimulate salivary flow, such as sugar-free lozenges and gums [29]. Acupuncture and hyperbaric oxygen (HBO) therapy have been also used as a possible intervention for the treatment of radiation-induced xerostomia in patients with a residual functional capacity of the salivary glands with controversial results [11, 30]. Low-level laser therapy (LLLT) has proven effects in promoting biomodulation in the cellular metabolism and has been effectively used as a salivary stimulants in patients with reduce salivary flow rate due to chemotherapy and radiotherapy [31]. More recently, salivary gland transfer and gene therapy, using human aquaporin-1 gene transfer, are strategies that appear potentially useful for preventing salivary gland radiation damage [32, 33]. The regenerative medicine options include adipose tissue–derived mesenchymal stem cell and adult salivary gland–derived stem cells [32].

### Graft vs. Host Disease- Induced SGD

Chronic graft vs.-host-disease (cGVHD) is a complication that may occur in 30–70% of patients undergoing allogeneic hematopoietic stem cell transplant (HSCT) [34]. Oral cGVHD is characterized by lichenoid changes, ulcers, erythema, and salivary gland hypofunction [35] Salivary gland involvement is characterized by destruction of secretory acini and ducts, resulting in decreased production of saliva and defense proteins [36]. HSCT patients typically experience dry mouth after receiving conditioning regimens, and this may persist through the period when salivary gland cGVHD develops, making the onset and diagnosis less evident. cGVHD of the salivary glands results in both quantitative and qualitative changes in salivary production, composition, and output [37]. Sialagogues such as pilocarpine and cevimeline have shown to improve symptoms in approximately two thirds of patients and recommended dosing is the same as described for RT-induced hyposalivation [38].

The effects of salivary gland hypofunction in these patients include rampant decay and recurrent oral candidiasis, especially if there is ongoing topical corticosteroid therapy for management of mucosal cGVHD, which suppresses mucosal immunity. An additional feature of oral cGVHD is the development of recurrent superficial mucoceles, suggesting that the underlying inflammation may be secondary to generalized mucosal involvement or because of direct salivary gland tissue targeting [39, 40].

### Chemotherapy- Induced SGD

Chemotherapy induced dry mouth is prevalent in 10–80% of patients undergoing treatment, regardless of the type of cancer [41]. The onset of oral symptoms generally begins in the 7th to the 10th day after the administration of chemotherapy and resolves after completion of chemotherapy, unlike radiotherapy-induced SGD, which persists for years after radiation therapy is completed. Symptoms include, but are not limited to, dry oral mucosa with consequential oral pseudomembranous candidiasis, halitosis, oral dysesthesia, hypogeusia, and difficulties in chewing, swallowing, and speaking [41].

Changes in salivary gland function can be caused by several chemotherapeutic agents including doxorubicin, cyclophosphamide, fluorouracil, methotrexate, and vinblastine [42]. Some cancer patients may also be on anticholinergic medications for therapy-induced nausea or diarrhea (e.g., for irinotecan-related early diarrhea) which may contribute to xerostomia [7].

Clinical presentation of chemotherapy induced SGD is variable, with some patients presenting with dry mucous membranes of varying severity, while others complaining of excessive salivation with drooling because of dysphagia or odynophagia. As opposed to RT-induced dry mouth, chemotherapy-related dry mouth is typically reversible. Therefore, management is palliative in nature and aims to relieve the temporary symptoms that manifest during treatment. This includes lifestyle modifications, use of saliva substitutes and mucosal lubricants, and short-term use of non-pharmacologic stimuli (gustatory stimuli, sugarless gum) [43, 44]. Acupuncture, tomotherapy, and 1% malic acid spray are currently under investigation, without published results yet.

### Immunotherapy–Induced SGD

Immune checkpoint inhibitors (ICIs) have proven to be an effective treatment option for patients with many forms of advanced cancers by preventing cancer cell evasion mechanisms through the suppression of major immune regulatory pathways, such as PD-1/PD-L1 and CTLA-4 [45]. The introduction of ICI therapy has been accompanied by a constellation of adverse events, likely secondary to T-cell–mediated immune reactions, resulting in increases in proinflammatory cytokines or enhancement of complement and autoantibody-mediated immune injury. Immune-related adverse events (irAEs) may virtually affect every organ system [46, 47], including the salivary glands. The development of ICI-induced sicca syndrome usually develops within the first 3–4 months of treatment [48]. Clinical features include severe hyposalivation with xerostomia, ocular dryness, and in some cases parotid gland swelling. Management depends on the severity of the irAE and is with over-the-counter topical agents, sialagogues, and systemic treatment with prednisone 1–2 mg/kg or hydroxychloroquine for severe cases [49]. ICI treatment interruptions may also help [50].

## SGD Complications and Management

### Dental Caries

Patient who develops dry mouth secondary to cancer therapy are at risk of developing dental caries. A literature review conducted by the Oral Care Study Group of the Multinational Association of Supportive Care in Cancer and the International Society for Oral Oncology (MASCC/ISOO) which included 37 head and neck cancer trials, showed that the prevalence of dental caries in head and neck cancer patients treated with radiation was 24% (4 studies) and 21% respectively (9 studies) for those receiving chemoradiation [51]. Patients with oral chronic GVHD also present with a > 50% increased number of cervical and interproximal dental caries [52]. The management and prevention of dental caries for cancer patients include regular dental care, maintenance of meticulous oral hygiene, daily sodium fluoride application for 3–4 min on teeth using a toothbrush or custom trays (patient should be instructed to avoid rinsing or eating for the next 30 min following the application) and dietary modification, minimizing consumption of cariogenic and acidic foods [53].

### Oral Candidiasis

Patients with salivary gland hypofunction may develop a secondary Candida infection in 39–62% of cases while receiving cancer treatment [54, 55] Although Candida albicans has been the most common Candida species detected in cancer patients, the prevalence of non-albicans Candida species (NACS) varies from 37% to 51% and is associated with increased drug resistance [56].Treatment of oral candidiasis involves either topical or systemic antifungal drugs; topical agents are considered preferable to systemic agents due to lower risk of side-effects and drug interactions [55]. The Infectious Diseases Society of America (IDSA) guidelines recommend the use of clotrimazole troches or nystatin suspension (easy to use for patient with hyposalivation) as first-line option for the management of mild candidiasis [57]. A common systemic agent as fluconazole is best used for short courses to prevent the development of resistance. Fluconazole was found to be effective in the prevention of clinical oral fungal infection and in the management of moderate to severe fungal colonization in patients receiving cancer therapy [57]. For fluconazole-refractory cases, the IDSA guidelines recommend the use of itraconazole or posaconazole, with voriconazole and amphotericin B and the echinocandins (caspofungin, anidulafungin, and micafungin) [55].

### Malnutrition

In cancer patients an average loss of 8 to 10% of body weight is common, even with early nutritional support [58]. SGD can contribute to difficulties in maintaining adequate nutrition during and after chemotherapy and RT. Cancer therapy may also cause dysphagia, odynophagia, loss of taste, dysgeusia and dehydration, all of which may result in malnutrition [59]. Patients on enteral nutrition had significantly less average weight loss during therapy (3.1 vs. 7.0 kg), required significantly fewer hospitalizations for dehydration and malnutrition, and had fewer interruptions in their cancer treatment (0 % vs. 18%) compared to patients on normal oral food intake [60, 61].

### Psychological Disorders

Individuals suffering from cancer are at high risk of experiencing major depressive episodes throughout treatment, although this risk appears to be especially prominent within the first year of diagnosis. The estimated prevalence of anxiety and depression among cancer survivors is 17.9% and 11.6%, respectively [62].

As part of the treatment regimen the most commonly prescribed medications include tricyclic antidepressants (TCAs) and selective serotonin reuptake inhibitors (SSRIs). While both classes of medications have been linked to hyposalivation, in a study done on parotid salivary flow rates, TCAs exhibited a 58% reduction in flow compared to 32% with SSRIs [63]. A meta-analysis performed by Capetta *et al*. further looked at selective norepinephrine reuptake inhibitors (SNRIs) and found that they significantly increased the risk of developing dry mouth when compared to SSRIs [64]. These data, when combined with the increased risk that head and neck cancer patients already face from SGD, indicate the need for careful prescription of antidepressants for this patient population [65].

## Conclusions

SGD is a common and debilitating complication that affects almost two-thirds of patients undergoing cancer treatment. Early diagnosis and management may result in decreased morbidity associated with SGD and improve well-being. Dental specialists are integral part of the cancer treatment team, and provide comprehensive education, supportive care, and the therapy of SGD related to cancer treatment. The current management approach of SGD is mostly palliative and aims to increase the amount of saliva and minimize the risk of secondary effects such as dental caries, dysgeusia and fungal infections.

Patients with SGD from cancer therapy should be followed by their dentist regularly for regular checkups and prescribed sodium fluoride 1.1% gel or toothpaste to reduce the risk of rampant decays.

In summary, SGD therapy is an important component of care prior to and during cancer therapy and amongst survivors. A multi-disciplinary approach is fundamental in the successful management of this complication in oncology and bone marrow transplant patients.

## Author Contributions

AVM is participated in conceptualization of the work, data collection and summary, and wrote the mini review. GP participated in data collection and preparation of the manuscript. VS provided feedback on the manuscript. CS participated as an advisor and in the manuscript preparation. AV supervised the work, participated in the analysis of the data, and manuscript preparation. All authors approved the final version of the manuscript.

## Conflict of Interest

The authors declare that the research was conducted in the absence of any commercial or financial relationships that could be construed as a potential conflict of interest.

## Publisher's Note

All claims expressed in this article are solely those of the authors and do not necessarily represent those of their affiliated organizations, or those of the publisher, the editors and the reviewers. Any product that may be evaluated in this article, or claim that may be made by its manufacturer, is not guaranteed or endorsed by the publisher.
